# Amino Acid Oncometabolism and Immunomodulation of the Tumor Microenvironment in Lung Cancer

**DOI:** 10.3389/fonc.2020.00276

**Published:** 2020-03-24

**Authors:** Johannes F. Fahrmann, Jody V. Vykoukal, Edwin J. Ostrin

**Affiliations:** ^1^Department of Clinical Cancer Prevention, University of Texas MD Anderson, Houston, TX, United States; ^2^Department of General Internal Medicine and Pulmonary Medicine, University of Texas MD Anderson, Houston, TX, United States

**Keywords:** lung cancer, tryptophan, asparagine, aspartate, glutamate, arginine, microenvironment

## Abstract

The observation that cancer acquires significant changes in its metabolism dates back nearly a century, to Otto Warburg noting that cancer cells preferentially utilize glycolysis even when there are no hypoxic conditions in the growth media. Altered energetics are thus considered a hallmark of cancer. However, it has become clear that altered metabolism is not limited to cellular energetic pathways. Alterations in amino acid synthesis and catabolism, lipid biogenesis, and other pathways such as polyamine processing are commonly seen in cancer. Additionally, alterations in metabolism do not only have profound effects for cancer cells but also affect their surrounding microenvironment. With new cancer therapeutics targeting the immune microenvironment, these effects may have implications on cancer growth and response to therapy. These interactions are profound in lung cancer, further demonstrating the manifold interactions between developing tumors and the inflammatory microenvironment. Here, we discuss how dysregulation of metabolism in cancer alters its microenvironment and how this newfound knowledge can be exploited for anticancer treatment.

## Introduction

As first reported by Warburg in 1923, cancer cells prefer glycolytic fermentation even in the presence of oxygen (1, 2). This widely observed effect was originally thought to be an underlying cause of cancer. However, this seems to be an epiphenomenon seen in many cancers from different tissues of origin which harbor a variety of driver mutations (3). While a driver mutation such as oncogenic KRAS directly alters metabolic pathways, cancers that successfully grow and evade the immune system undergo selective pressures from hypoxia, limited precursor availability, and secondary stimulation of pro- and anti-inflammatory responses by metabolic by-products (4–8). Given that metabolic pathways are tightly linked, alterations in one pathway have consequences in many others. The study of these changes, with the evolutionary processes that shape them and the cell-autonomous and non-autonomous effects of altered metabolism of cancer growth, forms a new field of oncometabolomics (9).

The Warburg effect is a common finding in cancer, and increased glucose utilization has been exploited to aid in detection, for instance, using fludeoxyglucose as a tracer in positron emission tomography (PET) scans (10). Research into the causes of altered energetics has led to tremendous insight about cancer biology, including forming the basis of an emerging field of mitochondrial dysregulation in cancer (11, 12). Energy coupling between tumor cells and their surrounding stroma has also come under close study. Tissue hypoxia and glycolysis lead to a buildup of lactic acid and thus an acidic, hypoglycemic microenvironment (8). This microenvironment has been shown to have a number of tumor-permissive and immune-suppressive effects, including promoting angiogenesis, invasion, and metastasis (13–15).

While the Warburg effect is a common observation in cancer cells, and glycolytic inhibitors are currently being studied as potential therapeutics, it is clear that there is significant metabolic plasticity and heterogeneity both within cancers and between cancer cells and their microenvironment. Many cancers continue to generate energy using standard oxidative phosphorylation in the mitochondria, even while utilizing the glycolytic Warburg pathway (16). Additionally, with cancer growth, there are dynamic changes in nutrient supply and oxygen concentration, and these are reflected in both cancer cell metabolism and cells composing the microenvironment. In what has been termed the “reverse Warburg effect,” cancer cells which still utilize oxidative phosphorylation can induce, through reactive oxygen species (ROS) in the microenvironment, surrounding stromal cells to upregulate glycolytic pathways. These pathways then cause by-products such as lactic acid to build up in the microenvironment and serve as fuel for the cancer's oxidative phosphorylative energy pathways.

Cancer cell energetics thus serve as a broader paradigm for oncometabolomics in a number of ways. Dysregulation is often a consequence of oncogenic mutation, but also may be a consequence of the evolutionary processes by which cancer cells overcome nutrient limitation and immune surveillance. Metabolic dysregulation within the cancer cells leads to profound changes both within cells and the microenvironment. While dysregulation of a metabolic pathway may not have a solitary cause, the same changes are observed frequently and thus serve as an enticing target for therapy. However, linkage between pathways, redundancy, heterogeneity, and plasticity may mean that metabolic inhibition may form only a piece in a larger puzzle. A similar example can be seen in folate and nucleobase synthesis pathways, which both support oligonucleotide synthesis and function as antioxidants to mitigate the effect of ROS. These changes may arise through oncogenic mutations such as those which activate the mammalian target of rapamycin (mTOR) pathway but also may represent the surviving cancer cells that can best support their unchecked cell division requirements and oxidative burden. These pathways are tied closely both to energetics and amino acid metabolism, and depletion of precursor molecules such as serine from the microenvironment has direct effects on T-cell function. Finally, this pathway is a classic example of how cancer metabolism can be targeted as antimetabolites targeting this pathway such as aminopterin, methotrexate, and fluorouracil were among the first chemotherapeutic agents to come into wide usage. However, these agents are commonly used as one part of a combination chemotherapy regimen, for instance, the combination of fluorouracil with irinotecan as current first-line therapy in metastatic colorectal cancer.

Examples of these highly interregulated pathways, with effects in the tumor cell and the microenvironment, are emerging throughout oncometabolomics. In lung cancer, there is a close relationship between developing cancers and the immune microenvironment, with both pro- and anti-tumor interactions changing dynamically through carcinogenesis (17–20). Here, we will detail examples primarily affecting pathways outside of cellular energetics, focusing on amino acid catabolism, and anabolism.

## Tryptophan Catabolism and the Immune Microenvironment

**Figure 1:**
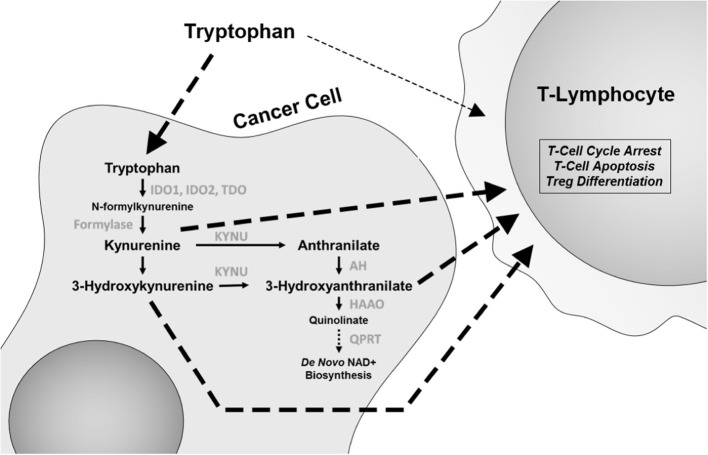
The tryptophan catabolic pathway. Changes in this pathway, including tryptophan depletion and accumulation of catabolites kynurenine, 3-hydroxyanthranilate, and anthranilic acid have immunomodulatory effects, with specific effects on CD8 T cells and regulatory T (Treg) cells. IDO1 and IDO2, indoleamine 2,3-dioxygenase 1 and 2; TDO, tryptophan 2,3-dioxygenase; KYNU, kynureninase; AH, anthranilate hydrolase; HAAO, 3-hydroxyanthranilate 3,4-dioxygenase; QPRT, quinolinate phosphoribosyl transferase.

Depletion of the essential amino acid tryptophan and accumulation of its catabolites including kynurenine (Kyn) lead to a microenvironment that is suppressive of an immune response and thus leading to promotion of tumor growth [Figure 1; (21, 22)]. In T cells, depletion of tryptophan and accumulation of Kyn directly induce differentiation into immunosuppressive regulatory T (Treg) cells and promote cell-cycle arrest and autophagy. This has led to intensive investigation of this pathway, including the development of inhibitors for the rate-limiting enzyme in tryptophan metabolism, indoleamine 2,3-dioxygenase (IDO) 1 and 2 and tryptophan 2,3-dioxygenase (TDO). Epacadostat is a small molecule inhibitor of IDO1 and IDO2, while indoximod is a tryptophan analog that affects multiple enzymes in the catabolic process.

Given the importance of T-cell infiltration and functionality in immune checkpoint blockade inhibitor (ICBI) therapy, initial trials have focused on these agents in combination with ICBI. While initial trials of epacadostat plus the programmed cell death protein 1 (PD-1) inhibitor pembrolizumab in metastatic melanoma were promising, a phase III, double-blinded, randomized trial (ECHO-301/KEYNOTE-252) of epacadostat/pembrolizumab vs. pembrolizumab did not note an additional benefit of epacadostat (23).

One possible explanation for this finding is that tryptophan metabolism may utilize multiple pathways. Epacadostat treatment led to only a 50% reduction in Kyn levels (24). Recent experimental evidence has demonstrated that TDO is equally effective in increasing Kyn levels in lung cancer (25). Moreover, the levels of IDO and TDO expression between tumor cells and mature dendritic cells in the microenvironment are quite disparate, with dendritic cells often expressing much higher levels than tumor cells. Additionally, other tryptophan catabolites in the Kyn pathway, such as 3-hydroxyanthranilate (3-HAA), have also shown to be immune suppressive, with direct effects on inhibiting T-lymphocyte activation and promoting Treg cell differentiation (Figure 1) and in non-antigen-stimulated T-cell proliferation (26, 27). Furthermore, *IDO1* expression is highly heterogeneous between different cancer types (28). As such, there is still a plausible potential role for pleiotropic tryptophan pathway inhibitors or a combination of inhibitors that act on multiple enzymes. To this end, a new concept has recently emerged wherein it is not IDO that is the active target, but rather the catabolism of Kyn through the administration of pharmacologically optimized PEGylated kynureninase (PEG-KYNase) (Figure 1). Specifically, treatment of tumor-bearing immune-competent mice with PEG-KYNase elicited statistically significant increases in tumor infiltration and expansion of CD8+ lymphocytes to elicit an anticancer effect (29). Importantly, PEG-KYNase when used in combination with checkpoint inhibitors or with a Gp96-Ig cancer vaccine yielded superior anticancer efficacy as compared to treatments alone (29). These findings highlight the potential of targeting other components of the Kyn pathway for the reversal of an immunosuppressive tumor microenvironment.

Aside from its role in modulating the tumor immunophenotype, recent work demonstrates a functional role of aberrant tryptophan metabolism in mediating resistance to chemotherapy (30). Specifically, cisplatin-resistant lung cancer cells were reported to exhibit increased consumption of extracellular tryptophan in comparison to parental lung cancer cells, and increased extracellular uptake of tryptophan was met with increased IDO1 activity through Kyn-mediated activation of the Aryl hydrocarbon Receptor (AHR) (30). Inhibition of IDO1 through pharmacological inhibition reduced the viability of cisplatin-resistant lung cancer cells via induction of increased generation of ROS (30). Notably, IDO-mediated AHR activation has been shown to induce interleukin (IL)-6-mediated activation of signal transducer and activator of transcription 3 (STAT3) signaling. Inhibition of IL-6 or STAT3 using siRNA and/or pharmacological inhibition reduced IDO gene and protein expression as well as Kyn formation, suggesting that IDO activity is sustained through an autocrine AHR–IL-6–STAT3 signaling loop (31). Consequently, these findings implicate cancer cell autonomous functions of aberrant tryptophan metabolism.

## Asparagine, Aspartate, and Glutamine in Cancer

In 1953, Kidd (32) described the antineoplastic effect of guinea pig serum, which, over the next 10 years, was found to be due to high levels of L-asparaginase. This enzyme catalyzes the degradation of the amino acid asparagine into aspartate and ammonia. Lymphoma and leukemia cell lines have a strong dependence for asparagine, and bacterially produced L-asparaginase was found to be highly active against multiple types of leukemia and lymphoma. It has been in wide clinical usage since the 1960s (33).

**Figure 2:**
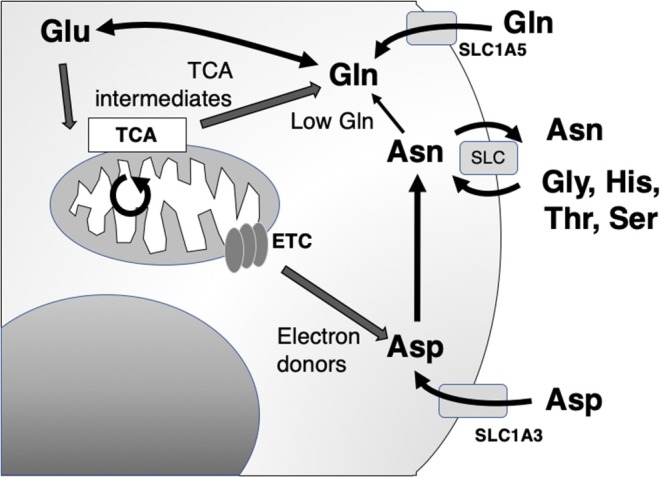
Asparagine, aspartate, glutamine, and glutamate shuttling to support mitochondrial and synthetic functions. Asn, asparagine; Asp, aspartate; Glu, glutamate; Gln, glutamine; Gly, glycine; His, histidine; Ser, serine; Thr, threonine; ETC, electron transport chain; TCA, citric acid cycle; SLC, solute carrier family.

The primary action of asparaginase is to reduce extracellular asparagine. It is most effective against cancers with low endogenous ability to synthesize asparagine *de novo*, as measured by the expression of asparagine synthetase (ASNS) and therefore requires importation of extracellular asparagine [Figure 2; (34)]. Asparagine synthesis is energy intensive and shunts vital resources, primarily the precursor glutamine (whose crucial roles are detailed below), toward asparagine synthesis. However, in the absence of glutamine, extracellular asparagine becomes essential as intracellular asparagine is shunted toward glutamine synthesis to prevent apoptosis (35, 36). Thus, the roles of intracellular vs. extracellular glutamine, asparagine, and the asparagine metabolite aspartate have come under close study.

The main role of intracellular asparagine is to serve as an amino acid exchange factor, with export of asparagine used to import other extracellular amino acids, particularly glycine, histidine, threonine, and serine [Figure 2; (37)]. Depletion of asparagine, for instance in a low ASNS-expressing cell, impairs import of amino acids and protein synthesis. Intracellular asparagine inhibits mTOR signaling, directly reducing protein initiation complex phosphorylation. This same mechanism also can upregulate nucleotide synthesis (36, 38).

A recently published study shows a role of asparagine in attenuating ATF4-mediated apoptosis during nutrient stress in response to KRAS signaling in non-small-cell lung cancer (NSCLC) (39). Specifically, glutamine-restriction induced KRAS-mediated upregulation of ATF4 through a KRAS–Akt–Nrf2–ATF4 axis. Induction of ATF4 led to increased expression of ASNS to sustain cellular proliferation and mitigate ATF4-mediated apoptosis as a consequence of nutrient deprivation. Targeting of asparagine through L-asparaginase manifests in near-complete stasis of tumors that were lacking ASNS, implicating both the necessity and targetable vulnerability of KRAS-driven ASNS activity during nutrient deprivation. To this end, the combination of MK-2206, an AKT inhibitor, in combination with L-asparaginase resulted in statistically significantly reduced tumor burden of H460 xenografts in comparison to control or single agent (39).

Aspartate is a non-essential amino acid in most cancer cells, and cells are able to replenish intracellular stores through *de novo* synthesis. Aspartate availability limits cancer cell growth through the control of nucleotide and protein synthesis. Interestingly, a major function of mitochondrial electron transport chain (ETC) function in cancer cells is to provide electron acceptors for robust activation of aspartate synthetic pathways (40, 41). Through this, aspartate becomes limiting for growth in hypoxia. Unlike asparagine, aspartate does not cross the plasma membrane readily, and in the setting of ETC inhibition, cells become dependent on upregulation of aspartate import from the microenvironment through upregulation of the transported Solute Carrier Family 1A3 (SLC1A3).

This functional relationship between intracellular and extracellular amino acid concentration has been most clearly described in glutamine metabolism, which is tightly linked to aspartate and asparagine (Figure 2). Several studies have provided experimental evidence that cancer cells are “glutamine addicted,” providing a source for carbon and nitrogen to replenish tricarboxylic acid (TCA) cycle intermediates and promote the biosynthesis of macromolecules, nucleotides, and diverse lipid species (42, 43). Extracellular uptake of glutamine is mediated by members of four amino acid transporter families. Among the four amino acid transporters, solute carrier family 1 member 5 (SLC1A5) has been shown to have the greatest affinity for glutamine (44). Although the biochemical fate of glutamine is diverse, in the context of cancer, it is often deaminated to glutamate through kidney-type glutaminase 1 (KGA) and GAC, a splice variant encoded by *GLS1* (42, 45–47). Glutamate, in turn, serves as a multifunctional metabolite, acting as a carbon donor for the TCA cycle intermediate α-ketoglutarate mediated by glutamate dehydrogenase, a substrate for glutathione biosynthesis and dioxygenases, as well as participating in an exchange with extracellular cystine via the cystine/glutamate antiporter, SLC7A11, to regulate intracellular redox balance (42, 48).

The interplay between glutamine metabolism and oncogenic signaling pathways has been previously described. For instance, oncogenic KRAS has been shown to reprogram metabolism by increasing the utilization of glutamine (49). Stable isotope tracer studies in KRAS mutant cancer cells have demonstrated that glutamine supports tumorigenesis by supplying carbon and nitrogen required for biomass synthesis (50). Moreover, recent studies have demonstrated that oncogenic KRAS signaling alters glutamine metabolism to support redox balance through increasing the reliance on transaminases (51). Similarly, GLS1 has been reported to be regulated by oncogenic c-MYC (52). Notably, MYC-driven GLS1 expression was not through direct transcription of the GSL1 gene by c-MYC, instead MYC-mediated suppression of GLS1 expression was mediated through targeting of the 3′ UTR of GLS1 by micro-RNAs miR-23a and miR-23b (52).

Although differential glutamine metabolism is a downstream consequence of oncogenic signaling pathways, such as KRAS or c-MYC, glutamine itself can also have direct effector functions. For example, glutamine has been linked to regulating the mechanistic target of rapamycin complex (mTORC1) activity through the efflux of essential amino acids, such as leucine, into cells via SLC7A5/SLC3A2 bidirectional transporters (53). Notably, authors found that leucine disrupts the Sestrin2-GATOR2 interaction, resulting in mTORC1 translocating to the lysosome where Rheb-GTPase subsequently enhances mTORC1 activity (54, 55). Moreover, glutamine-stimulated mTORC1 activity has been shown to impair autophagy initiation through the negative regulation of ULK1 or through elimination of ROS by glutathione and nicotinamide adenine dinucleotide phosphate (NADPH) (56–58). Consequently, there is considerable interest toward anticancer targeting of glutamine metabolism, namely, through inhibition of glutaminase activity.

To date, there are three commonly explored inhibitors of glutaminase: (5-(3-bromo-4-(dimethylamino)phenyl)-2,2-dimethyl-2,3,5,6-tetrahydrobenzo[a]phenanthridin-4(1H)-one) (glutaminase inhibitor 968), Bis-2-(5-phenylacetamido-1,3,4-thiadiazol-2-yl)ethyl sulfide (BPTES), and telaglenastat (CB-839). Glutaminase inhibitor 968 is an allosteric inhibitor of KGA and GAC (59), whereas CB-839 and BPTES are non-competitive selective inhibitors (46, 60). Several lines of evidence have demonstrated utility of glutaminase inhibitors as anticancer agents in a variety of cancer types including breast cancer (46, 61), lung cancer (62), pancreatic cancer (63), melanoma (64), and hematological malignancies (65). Moreover, several studies have demonstrated that inhibition of glutaminase increases the radiosensitivity and chemosensitivity of cancer (46, 66–68).

While glutamine is an important metabolite readily scavenged by cancer cells, it also plays an additional role in the tumor microenvironment by serving as an important biomolecule for immune cells, particularly T cells. In active T cells, increased glutaminolysis supports T cell function by providing carbon and nitrogen for proliferation-associated biosynthetic pathways (69). For example, it was reported that glutamine-derived α-ketoglutarate regulates CD4+ T cell differentiation by increasing the expression of the gene encoding the T helper 1 (Th1) cell-associated transcription factor Tbet to promote differentiation into Th1 T cells. Upon glutamine restriction, CD4+ T cells, when activated in the presence of cytokines that promote Th1 differentiation, instead differentiated into FOXP3+ Treg cells (69). Others have reported similar findings that glutamine restriction drives CD4 T cells toward FOXP3+ Treg cells by reducing *de novo* nucleotide synthesis and increasing reliance on generation of endogenous glutamine via glutamine synthetase (GS). Blocking of GS reduced FOXP3+ Treg cell proliferation and maintenance under glutamine restriction (70).

Collectively, these studies suggest that targeting glutamine metabolism in tumors may attenuate glutamine depletion in the local tumor microenvironment and enable a more potent antitumor immune response. To this end, previous studies in a CT26 colon cancer syngeneic mouse model demonstrated that the combination CB-839 plus anti-PD-1/anti-PD-L1 yielded superior anticancer efficacy compared to either treatment alone (71). More recently, Calithera initiated a phase 2 study to evaluate the safety, tolerability, and efficacy of CB-839 in combination with the PD-1/programmed cell death protein ligand 1 (PDL-1) immune checkpoint inhibitor nivolumab in patients with solid tumors. Reported results demonstrated acceptable toxicity with mild to moderate adverse events; overall response rates of 19% and an overall disease control rate of 44% were reported in patients with melanoma. (Calithera; Society for Immunotherapy of Cancer Meeting) A trial of CB-839 in combination with the third-generation epidermal growth factor receptor (EGFR) inhibitor osimertinib in EGFR-mutated NSCLC is also currently enrolling (ClinicalTrials.gov NCT03831932).

## Arginine Metabolism

Arginine has multiple metabolic fates depending on cell type, developmental stage, and state of health or disease. Normally, a semi-essential amino acid in humans and most other mammals, endogenous arginine synthesis proceeds primarily through the “intestinal–renal axis”: citrulline precursor derived from glutamine, glutamate, or proline in the intestine is converted to arginine in the proximal tubule cells of the kidney (72). Elevated arginine requirements of carcinoma cells *in vitro* were first reported over 70 years ago (73). Many cancers, in particular, lymphomas, melanoma, mesothelioma, and hepatocellular, renal cell, and prostate carcinomas that are otherwise chemoresistant and with poor clinical outcome, exhibit decreased expression of arginine metabolizing enzymes including argininosuccinate synthetase (ASS1) and/or ornithine transcarbamylase (OTC) and are thus auxotrophic for arginine (74). Deprivation of circulating arginine via enzymes such as arginase, arginine deiminase (ADI), and arginine decarboxylase (ADC) exploits a significant metabolic vulnerability of cancer cells in these tumor types, and such enzyme-mediated arginine depletion is currently under clinical investigation along multiple fronts to advance this potential class of anticancer therapeutics (75–77). While positive results have been reported in this regard, the role of arginine in tumors in general is multidimensional. In addition, resistance to arginine depletion therapy has been observed to be overcome by ASS1 reexpression in previously sensitive cells via c-Myc activation of the ASS1 promoter (78).

In contrast to arginine auxotrophs, many epithelial tumor types overexpress ASS1, particularly those that are characterized by moderate to high *de novo* sensitivity to platinum chemotherapy (79). These include primary epithelial ovarian cancers, as well as stomach, lung, and colorectal cancers. Of particular interest is an emerging insight into the role of arginine in modulating inflammatory networks and immune cell reactivity within the tumor microenvironment. Proinflammatory cytokines tumor necrosis factor (TNF)-α and IL-1β regulate ASS1 in cancer cell lines, and TNF-α has been observed to co-localize with ASS1 in epithelial ovarian cancer (80, 81). In addition, ASS1 intersects with a number of metabolic and inflammatory pathways, and cytokines such as TNF-α modulate key enzymes downstream of ASS1, including nitric oxide synthases (NOS) and ornithine decarboxylase (ODC) (82). Downstream metabolites of arginine such as ornithine and citrulline are evidenced to affect T-cell activation, and thus, dysregulated arginine metabolism in cancer appears to modulate innate and adaptive immunity to promote tumor survival and growth. A better understanding of this intersection between cancer cell metabolic reprogramming, oncometabolites, and immune response across different cancer types is essential to overcoming immune escape and increasing the efficacy of immunotherapeutic intervention (83).

## Conclusions

Metabolic alterations in cancer are not only a consequence of oncogenic mutation or of evolution through nutrient limitations. Rather, there is a complex interplay of metabolic precursors and by-products between a cancer cell and its surrounding environment. Recent advances highlight that these metabolic shifts modulate not only tumor growth but the host immune and microenvironmental response. Given the profound effect of these pathways in lung cancer, these pathways hold tremendous potential for biomarker development and intervention.

## Author Contributions

EO, JF, and JV prepared and reviewed the manuscript.

### Conflict of Interest

The authors declare that the research was conducted in the absence of any commercial or financial relationships that could be construed as a potential conflict of interest.
